# 4321 Personalization of T cell production for cellular immunotherapy

**DOI:** 10.1017/cts.2020.88

**Published:** 2020-07-29

**Authors:** Dennis Jinglun Yuan, Shuai Shao, Joanne H Lee, Stacey M Fernandes, Jennifer R Brown, Lance C Kam

**Affiliations:** 1 Columbia University; 2 Dana Farber Cancer Institute; 3 Harvard Medical School

## Abstract

OBJECTIVES/GOALS: Utilize polymer-based fiber scaffolds and machine learning methods applied to patient biomarker data to enhance and personalize T cell expansion and production for T cell therapy in chronic lymphocytic leukemia. METHODS/STUDY POPULATION: Scaffolds are 1) generated from a co-polymer blend of PDMS and PCL with controlled fiber diameters and pore size, 2) coated with activating antibodies to CD3 and CD28, and 3) used to stimulate T cells from both healthy donors and CLL patients. CLL patients have pre-annotated mutation burdens and clinical biomarkers. T cell populations will be analyzed for exhaustion markers and phenotypes before, during, and after expansion. Cell functionality will be measured by cytokine secretion, cell cycle analysis, and fold expansion, with respect to platform parameters, and analyzed with inputs of disease markers and exhaustion profile of isolated T cells using regression and random forest classifiers. RESULTS/ANTICIPATED RESULTS: We previously showed that engineering the mechanical rigidity of activating substrates can enhance and rescue T cell expansion from exhausted populations. Now we aim to study a broader range of compositions and geometry of scaffolds with respect to capacity to expand CLL T cells. Preliminary data with fiber diameters ranging from 300 nm to 6 um confirm the effect of geometry in modulating expansion. A biorepository of T cells from 80 CLL patients have been isolated concurrently. Anticipated results include correlating exhaustion profile of T cells with clinical biomarkers and identifying markers associated with expansion on panel of platform parameters. DISCUSSION/SIGNIFICANCE OF IMPACT: T cell therapy has shown particular promise in treating blood cancers, yet significant percentage of T cells isolated from patients undergoing treatments are unresponsive to activation. A powerful tool is to predict if and how patient T cells can be robustly expanded on a personalized approach.

